# Kisspeptin Variations in Patients with Polycystic Ovary Syndrome—A Prospective Case Control Study

**DOI:** 10.3390/medicina58060776

**Published:** 2022-06-08

**Authors:** Mona Akad, Răzvan Socolov, Cristina Furnică, Roxana Covali, Catalina Daniela Stan, Eduard Crauciuc, Ioana Pavaleanu

**Affiliations:** 1 Department of Obstetrics and Gynecology, University of Medicine and Pharmacy “Gr. T. Popa”, 700115 Iași, Romania; akad.mona@yahoo.com (M.A.); cristina.furnica@umfiasi.ro (C.F.); rcovali@yahoo.com (R.C.); catalina.stan@umfiasi.ro (C.D.S.); crauciuc@yahoo.com (E.C.); ioana_pavaleanu@yahoo.com (I.P.); 2 Clinical Hospital of Obstetrics and Pharmacy “Elena Doamna”, 700398 Iași, Romania

**Keywords:** kisspeptin, polycystic ovary syndrome, infertility

## Abstract

*Background and objectives*: Kisspeptin, also named metastin, showed important roles in initiating the secretion of gonadotropin-releasing hormone (GnRH) and is an essential factor in the development of polycystic ovaries syndrome (PCOS). Several research studies noticed associations between kisspeptin levels and patients with anovulatory cycles due to PCOS with an increased LH/FSH ratio. The aim of our study was to bring scientific evidence regarding the correlation between high kisspeptin and luteinizing hormone values in subfertile women due to PCOS. *Materials and Methods:* A prospective case-control study was conducted in “Elena Doamna” Hospital of Obstetrics and Gynecology between 4 January 2021 and 1 March 2022. All patients agreed to participate in our study, had ages between 18 and 45 years old, and had a body mass index between 18.5 and 30 kg/m^2^. The study group consisted of subfertile patients with PCOS and menstrual disturbances, including amenorrhea or oligomenorrhea. The control group consisted of healthy patients with ovulatory cycles and no other reproductive or endocrinology pathologies. During the follicular phase of their menstrual cycle, patients had blood samples taken with the dosage of kisspeptin, LH, FSH, estradiol, insulin, glycemic levels, testosterone, and prolactin. Pelvic ultrasounds and clinical examinations were performed as well. *Results*: Significant differences were observed in kisspeptin, LH, FSH, and estradiol levels between patients with PCOS and the control group. After the univariate analysis, PCOS was significantly associated with increased kisspeptin, increased LH, and decreased FSH. There was no significant association between PCOS, estradiol, prolactin, and insulin. *Conclusions*: kisspeptin serum values are higher in subfertile PCOS patients, supporting the hypothesis that an over-stimulation of the KISS1 system might cause the hyper-stimulation of the HPG-axis.

## 1. Introduction

Kisspeptin, formerly known as metastin, was discovered in 1996 in Hershey, Pennsylvania. The gene was called KISS1 as a tribute to the city where it was studied for the first time. Kisspeptins represent proteins encoded by the KISS1 gene in human species and form ligands of the G-protein coupled receptor, GPR54. It was initially named metastin due to the capacity to suppress cancerous cells in melanoma and breast cancer metastasis [1]. Further studies showed important roles of the kisspeptin-GPR54 system in initiating the secretion of gonadotropin-releasing hormone (GnRH) during puberty [2]. The KISS1 gene located on human chromosome1q32 encodes the kisspeptin precursor, a peptide composed of 145 amino acids, but numerous forms have been discovered since. Kisspeptin-54 is the major fragment, structured with the help of 54 amino acids. Kisspeptins with lengths of 14, 13, and 10 amino acids have also been identified and are the subject of ongoing research [3].

KISS1 binds to its specific receptor, GPR54, or lately called KISS1R, and activates the G-protein and phospholipase-C and further on phosphatidylinositol bisphosphate leads to diacylglycerol and inositol trisphosphate, resulting in elevated levels of calcium, closure of potassium channels, opening of cation channels, and depolarization of GnRH neurons. As a consequence of depolarization, GnRH neurons start the hormonal secretion [4]. 

Administration of kisspeptin antagonists were able to suppress GnRH pulsatility in animals, implying that actually kisspeptin neurons are responsible for the GnRH pulse generator [5,6]. Experimental studies performed on human species proved that the administration of kisspeptin induces an elevation of LH levels in female patients [7]. Human subjects identified with KISS1R mutations and hypogonadotropic hypogonadism (HH) responded to GnRH administration. According to recent studies, we can affirm that kisspeptins are without doubt excitatory stimulants for the GnRH in the hypothalamic-pituitary-gonadal (HPG) axis [8]. While kisspeptin is able to increase levels of LH, its effects on the follicle-stimulant hormone (FSH) are delayed and less expressed. Fewer effects on FSH might be due to differences in secretory patterns of gonadotrophins or different responses to kisspeptins [9]. According to Jayasena et al.’s study performed in 2014, the administration of kisspeptin lead to an over 2-fold increase in LH levels and a small to non-existent increase in FSH levels [10]. Several research studies noticed that kisspeptin administration induces a direct effect on the upstream regulation of GnRH neurons when it comes to the depolarization process and an up-regulated expression of the GnRH mRNA, therefore leading to an increased LH/FSH ratio. 

Polycystic ovary syndrome (PCOS) is one of the most common endocrine pathologies encountered bt female patients at reproductive age and the most frequent cause of infertility due to anovulation. According to Rotterdam’s criteria from 2003, the diagnosis of PCOS is established when two out of three or all criteria are met: chronic anovulation with oligo/amenorrhea, clinical features or biochemical evidence of high testosterone levels, and polycystic ultrasound appearance of the ovaries [11]. 

PCOS can be classified into four main phenotypes according to the features used for the diagnosis criteria. Patients with phenotype A have hyperandrogenism, ovulatory dysfunction, and polycystic ovarian morphology, whereas patients with the B phenotype have ovulatory dysfunction and hyperandrogenism. A third category of patients with PCOS categorized as phenotype C have hyperandrogenism and polycystic ovarian morphology. Phenotype D consists in patients with ovulatory dysfunction and polycystic ovarian morphology [12]. In 2018, the international evidence-based guideline for the diagnosing and management of patients with PCOS sustained and endorsed the classifying of PCOS into four phenotypes [13]. 

Associations between kisspeptin levels and patients with anovulatory cycles due to PCOS represents a new and prosperous research area for doctors working in the assisted-reproductive-techniques field. Considering the relationship between HPG axis and kisspeptin, this study was performed to measure kisspeptin serum levels in patients with PCOS and correlate kisspeptin levels with female reproductive hormones and body mass index (BMI).

## 2. Materials and Methods

Our study was performed in “Elena Doamna” Clinical hospital of Obstetrics and Gynecology—Iasi between 4 January 2021 and 1 March 2022. It was a prospective case-control study and included two groups of patients. The study group consisted of female patients with ages between 18 and 45 years old, a body mass index between 18.5 and 30 kg/m^2^, and a diagnosis of PCOS according to Rotterdam criteria. Patients with other endocrine pathologies aside from PCOS that might have interfered with our hormonal laboratory exams or clinical examinations were excluded. All patients agreed to enter our study and signed the consent form before being examined. 

The control group consisted of female patients with ages between 18 and 45 years old, BMI between 18.5 and 30 kg/m^2^, and having no history of PCOS or other endocrine pathologies that might have influenced results. 

Exclusion criteria consisted in patients that did not have the appropriate age or BMI; suffered from endometriosis, autoimmune diseases, or thyroid pathologies; had prolactinomas, Cushing’s disease, or other potential causes for anovulation and/or hyperandrogenemia; were under hormonal treatment; or had given birth 12 months prior to entering our study inability to follow-up.

All patients that agreed and signed the consent form went through a thorough clinical examination. During clinical examination, weight and height, signs of hirsutism, acanthosis nigricans, facial or body acne, or menstrual cycle irregularities were noted. The anamnesis and medical history were noted in detail in the patient’s chart and recorded for the study as well. During the follicular phase of the menstrual cycle, patients were recalled to our unit in order to take blood samples and perform a pelvic scan. The laboratory exams consisted in the dosage of kisspeptin, LH, FSH, estradiol, insulin, glycemic levels, testosterone, and prolactin. For these tests, five milliliters of blood were taken from the participants. After applying all inclusion and exclusion criteria, we obtained a total number of 61 patients: 24 patients included in the control group and 37 patients included in the study group. Patients included in the control group were fertile patients with regular menstrual cycles. The 37 infertile patients were diagnosed with polycystic ovary syndrome, and the diagnosis was based on the Rotterdam criteria in 2003. Menstrual disturbances included amenorrhea marked by the absence of menstrual cycles for more than 6 months and oligomenorrhea marked by a delay in menses of more than 35 days for 6 months. The ultrasound examination using the transvaginal probes was performed once for all patients. 

The test protocol included obtaining 5 milliliters of blood once from all patients; samples were taken during one of their first 3 days of menstruation. All samples were incubated at room temperature for two hours; next, the serum was separated by centrifugation for 20 min at 3000 rpm. Further on, all samples were transferred to plain tubes and stored at 20 degrees Celsius until the assay process. All parameters were measured by enzyme-linked immunosorbent assay (ELISA).

In this study, the values are presented as mean ± SD, and a one-way analysis of variance (ANOVA) was performed to estimate the difference between the groups. 

## 3. Results

**Figure 1:**
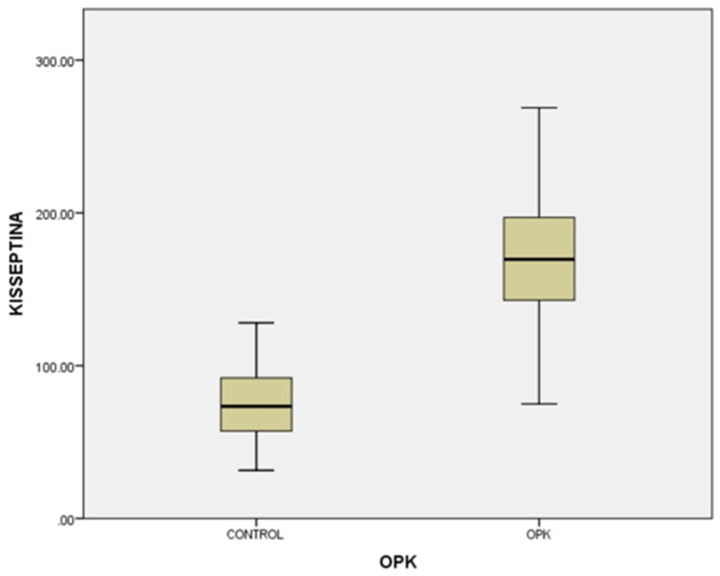
Univariate analysis of the relationship between kisspeptin serum levels and PCOS (*p* < 0.001).

**Figure 2:**
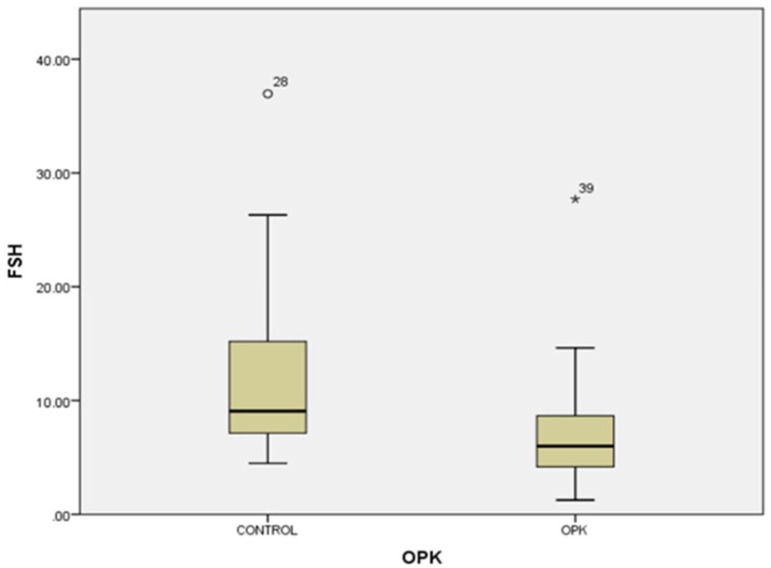
Univariate analysis of the relationship between FSH serum levels and PCOS (*p* < 0.005)**.** Circles represent high potential outliers and the stars represent high extreme values.

**Figure 3:**
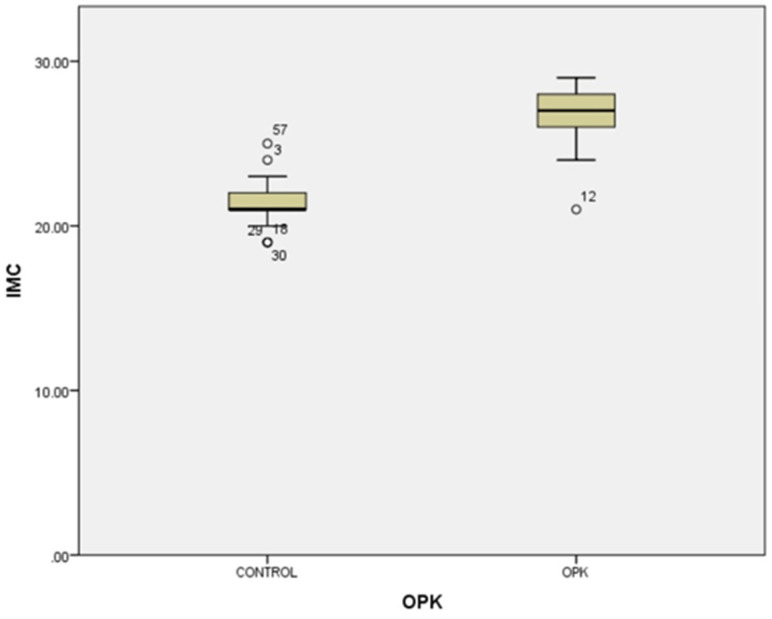
Univariate analysis between body mass index and PCOS (*p* < 0.01). Circles represent high potential outliers and the stars represent high extreme values.

**Figure 4:**
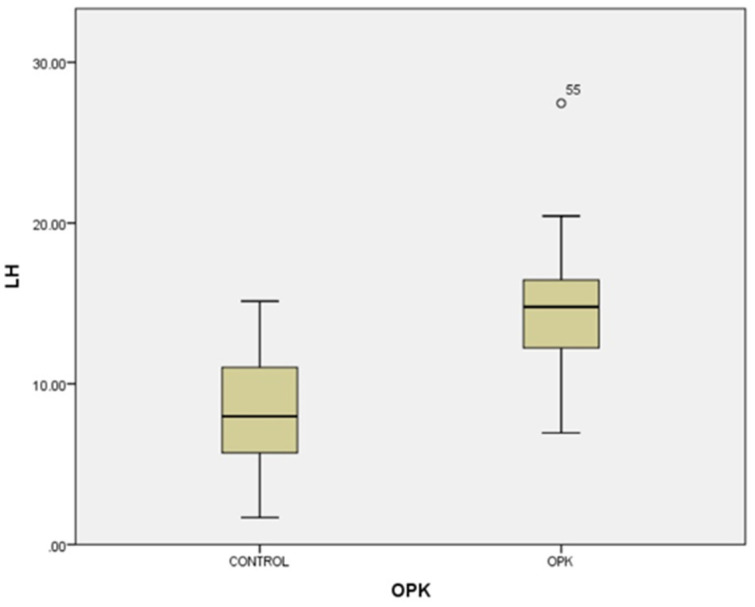
Univariate analysis between LH serum levels and PCOS (*p* < 0.001). Circles represent high potential outliers and the stars represent high extreme values.

In our study, the cases and controls had similar general medical data. Significant differences were observed in kisspeptin, LH, FSH, and estradiol levels between patients with PCOS (subfertility) and the control group (healthy patients). After the univariate analysis, PCOS was significantly associated with increased kisspeptin (*p* < 0.001, 95% CI 7.5–11.504) (Figure 1) and decreased FSH (*p* < 0.005, 95% CI 1.90–8.56) (Figure 2). We also found that BMI correlates positively with PCOS (*p* < 0.01) (Figure 3) and PCOS correlates with increased LH (*p* < 0.001, 95% CI 4.10–7.97) (Figure 4), There was no significant association between PCOS, estradiol (*p* = 0.7), prolactin (*p* = 0.13), and insulin (*p* = 0.41) (Table 1). There was a significant correlation between increased kisspeptin and increased LH in PCOS patients (*p* < 0.001, 13.01 95% CI 10.77–13.25) but not with FSH, estradiol, prolactin, and insulin. 

## 4. Discussion

The recent discovery of KISS1/KISS1R and its effects on upregulating GnRH has created a new pathway for further investigations considering patients with PCOS. New studies have brought clear evidence about the presence of kisspeptin at the ovarian level and the way it is involved in the ovulation process and sex hormone regulation. PCOS is a heterogeneous syndrome; therefore, it is possible that not all patients share the same endocrine disturbances and hormonal alterations [14]. 

Neurons responsible for the secretion of kisspeptin are located mainly in two areas: the hypothalamic arcuate nucleus (ARC) and the preoptic area. Steroid sex hormone concentrations are responsible for the kisspeptin modulation of GnRH secretion. Studies performed on laboratory animal models have proven that kisspeptin neurons modulate the periovulatory positive estrogen feedback on GnRH [15]. It has been considered that during the periovulatory period, the pulsatile secretion of GnRH is influenced by kisspeptin’s regulatory effects on neurons. The pulsatile GnRH secretion induced by kisspeptin stimulates the release of both gonadotropins in a regular manner from the pituitary. Negative feedback caused by estradiol levels during the follicular phase and due to progesterone during the luteal phase are mainly exerted through particular effects on ARC kisspeptin neurons [16] even though, when regarding the reproductive axis in human species, not all neurons responsible for GnRH secretion are connected to neurons responsible for kisspeptins secretion [17]. When reconsidering the physiological effects of kisspeptin on LH secretion induced by GnRH neurons, there is obvious confirmation of the hypothesis that PCOS patients have an imbalanced kisspeptin secretion [18]. PCOS represents a complex and multifactorial syndrome due to abnormal neuroendocrine secretion and metabolic impairments.

An exact cause for the appearance of PCOS was not identified, but patients with PCOS often show a neuroendocrine disturbance reflecting a perturbation of the GnRH neuronal activity and increased LH pulsatility [19].

High LH pulsatility contributes to an increased thecal androgen secretion and incapacity to release the oocyte, therefore leading to anovulation (Marshall et al., 2001; Caldwell et al., 2017). Kisspeptin and neurokinin B (NKB) pathway have been pointed out as main regulators of GnRH and LH secretion (Pinilla et al., 2012; Skorupskaite et al., 2014; Clarkson et al., 2017). Patients with mutations on KISS1/KISS1R and neurokinin B showed delayed puberty (de Roux et al., 2003; Seminara et al., 2003; Topaloglu et al., 2009; Topaloglu et al., 2012), while patients with precocious activating mutations on showed precocious puberty (Teles et al., 2008). According to a randomized controlled trial, administration of neurokinin 3 receptor antagonists in women diagnosed with PCOS showed a decrease in the frequency of LH pulsatility (George et al., 2016). Taking into consideration the results of these recent studies, it is possible that a dysregulation of the NKB signaling may be the culprit in the neuroendocrine pathogenesis of PCOS [20]. According to Skorupskaite et al., in patients with PCOS, kisspeptin antagonists may help in reducing high LH values and restore ovulation and follicular development [2].

A study concerning patients with infertility included a group of females with PCOS and discovered that measured kisspeptin levels were significantly higher when compared with their other two groups with infertility due to male-factor infertility and unexplained infertility. It was suggested in the conclusions that increased kisspeptin levels could represent a reliable marker to estimate the antral follicle count and future criteria for diagnosing PCOS [21].

In this study, kisspeptin levels during follicular phase of the menstrual cycle were significantly higher in PCOS patients suffering from primary or secondary infertility when compared to the control group. Studies performed by Yarmolinskaya et al. in 2017 and Nyagolova et al. in 2016 were in agreement with our results by showing high serum kisspeptin levels in PCOS patients during their follicular phase [22,23]. Studies trying to assess kisspeptin levels in association with the PCOS phenotype have been taken in consideration. Some studies performed on rodents managed to provide evidences of certain values of serum kisspeptin for each PCOS phenotype, but none of the existing studies evaluated the kisspeptin values in each PCOS phenotype. 

According to Aliabadi et al. [24], in female rats that received letrozole injections, kisspeptin-positive cells levels and expression of mRNA were higher than in the control group of normal female rats. They also proved that in PCOS rats, enhanced neural cells might be the origin of LH hypersecretion. On the contrary, Marcondes et al. [25,26] suggested that in rats treated with testosterone, low kisspeptin expression might be a cause for anovulation and decrease in LH secretion. Inconsistencies of the results regarding the androgenic effects of kisspeptin are due to different research methods used. In all previous studies performed on rats that identified high serum levels of kisspeptin in association with PCOS, they observed that these high levels were associated with PCOS phenotypes with high LH levels. These results are in line with our hypothesis that kisspeptin serum levels may vary among PCOS phenotypes.

In the present study, we found that serum levels of kisspeptin were higher in patients with higher BMI when compared to patients with normal BMI. Our results are in opposition with findings of Rafique and Latif [27], who obtained the same kisspeptin serum levels in normal and overweight patients; their study was performed on groups of female patients originating from Saudi Arabia. On the contrary, Koloziejski et al. [28] identified increased kisspeptin levels in patients with normal BMI when compared to overweight patients. Our study included patients with a BMI lower than 30 in both groups. Variations of kisspeptin values in correlation with BMI might be normal, considering most studies are performed on different ethnic populations, and our study took into consideration only female patients originating from Romania. 

We identified no statistical differences in serum kisspeptin levels in different ages in PCOS patients. Our findings are in accordance with studies performed by Jeon et al., Gorkem et al., and Emekci Ozay et al. [29,30,31], which did not observe any correlation between kisspeptin serum levels and age in PCOS patients. 

A group of female patients with functional hypothalamic amenorrhea caused by low body weight received, during a trial study, injections with kisspeptin-54. Results showed that kisspeptin-54 has the ability to stimulate gonadotropin secretion; effects were significant after the first shot but reduced in intensity after being administered for two weeks [32]. When changing the way of administration from twice a day to twice a week, the secretion of gonadotropins was sustained [33]. 

Another study conducted by Ruka et al. in 2013 showed that the amount of KISS1 and kisspeptin cells in the arcuate nucleus positively correlates with LH levels in rodent subjects with PCOS [34]. Neurokinin B (NKB) stimulates kisspeptin neurons via specific receptors, and further on, kisspeptin stimulates GnRH neurons, therefore suggesting a role of ARN KNDy (kisspeptin, neurokinin B, dynorphin) neurons in initiating GnRH hyperactivity and downstream elevated LH pulse secretion. Through a clinical study targeting PCOS women, it was attempted to modulate the KNDy neurons and decrease their activity in order to reduce LH secretion. Patients were administered an antagonist for neurokinin B receptor for 28 days, and results showed significant reduction of LH pusatility [35]. 

One of the main causes of infertility nowadays is represented by endometriosis. Even though multiple studies have been performed in order to establish a correlation, results were often inconsistent. Timologou et al. nevertheless managed to correlate significantly higher levels of kisspeptin in endometriosis lesions when compared to eutopic endometrium, suggesting that KISS1 might have a role in the pathogenesis of endometriosis [36]. 

Potential diagnostic and therapeutic applications for kisspeptin antagonists and agonist have been discovered. For patients suffering from infertility, the use of kisspeptin agonists may localize lesions in the HPG axis disturbance and can be used as a method of evaluation for the gonadotrophic potential. Their ability to stimulate the LH secretion may represent a method of inducing ovulation in patients with subfertility. On the other hand, the use of kisspeptin antagonists is a way of reducing the LH pulse frequency and amplitude without harming the basal secretion of LH. Using kisspeptin antagonists as contraceptives in female patients or therapeutically in sex-steroid-dependent pathologies, such as breast cancer, endometriosis, uterine fibroid, or prostate cancer, represents an essential subject to be investigated [37]. Nakamura et al. studied KISS1-knockout rats and managed to prove that they exhibited a lack of both pulse and surge modes of gonadotropins (both LH and FSH) secretion and failure of puberty development, indicating that kisspeptin has an indispensable function in the cyclic secretion of GnRH in order to regulate normal reproductive function and puberty onset. The male knockout mice had no male sexual behavior and showed female-like reflexes for lordosis, suggesting that kisspeptin is essential for the masculinization of sexual behavior control in male rats [38].

One of the four experimental studies performed on monkeys by Kathryn et al. in 2012 was meant to determine whether there are changes in the GnRH secretion when kisspeptin antagonists are administered. Laboratory subjects were administered with the kisspeptin antagonist peptide 234 or a vehicle (perfusion fluid) through microdialysis probe for 30 min. Five prepubertal and four puberal monkeys were used in this study. Results showed that peptide 234 managed to suppress the GnRH secretion in prepubertal monkeys in a significant manner when compared to monkeys that had perfusion fluid administered. The GnRH secretion was suppressed during the administration and up to 90 min after the start of the infusion. The same situation was encountered in pubertal monkeys with the difference that GnRH suppression was mostly identified during the suppression and not afterwards [39]. 

Studies have focused recently on the use of gonadotropin-inhibitory hormone (GnIH) in order to reverse or improve the PCOS-like features. In 2019, Anushree et al. administered GnIH and GnIH antagonists (RF-9) to PCOS mice for a period of 8 days. Their results to this specific exogenous treatment managed to reduce the body weight, decrease low-density-lipoproteins cholesterol concentrations (LDL) levels, and decrease the serum testosterone levels as well. Fertility, on the other hand, was not restored, as under treatment, mice failed to ovulate and develop large, healthy antral follicles or the appearance of a functional corpus luteum [5]. It was showed that GnIH has suppressing effects on kisspeptin-induced GnRH release in hypothalamic areas in mice. This process was discussed, and it might result from the inhibition effects of GnIH on the exocytosis of GnRH and not on the KISS1/KISS1R complex. Considering the implication and the hyper-stimulation culpable for the development of PCOS, we should concentrate our studies on the use of GnIH as a possible novel therapeutic solution for patients with infertility due to PCOS [40].

Even though most investigations concerning kisspeptins have been concerning infertility and reproductive pathologies, for the first time, it was discovered as a metastasis suppressor [1]. Suppressing of the metastasis is done by restricting the growth of secondary tumors [41]. Connecting kisspeptin to its receptors elevates intracellular levels of calcium and activation of MAPKs, therefore limiting cellular proliferation and motility [42].

## 5. Conclusions

In conclusion, kisspeptin serum values are higher in subfertile PCOS patients, supporting the hypothesis that an over-stimulation of the KISS1 system might cause the hyper-stimulation of the HPG-axis, therefore leading to consequences such as excessive testosterone secretion and irregular menstrual cycles. Results from studies done on laboratory rats imply that kisspeptin levels are not increased in all subtypes of PCOS. In our study, we conclude that kisspeptin values are elevated in PCOS associated with higher LH levels and high BMI.

Future studies researching the relationship between kisspeptin and metabolic and endocrine disorders, such as insulin resistance, are needed.

## Figures and Tables

**Table 1:** Univariate analysis between presence of PCOS and reproductive hormones levels during follicular phase of the menstrual cycle.

Hormone	Mean Value PCOS Group	Mean Value Control Group	*p*	95% CI
FSH	6.949 mUI/mL	11.834 mUI/mL	0.005	1.90–8.56
LH	14.393 mUI/mL	8.343 mUI/mL	0.001	4.10–7.97
Estradiol	74.973 pg/mL	69.565 pg/mL	0.7	−34.159–24.58
Prolactin	621.679 µUI/mL	501.829 µUI/mL	0.13	19.38–19.50
Insulin	9.265 µUI/mL	10.485 µUI/mL	0.41	−1.62–4.47
Kisspeptin	130.499 pg/mL	76.243 pg/mL	0.001	7.55–11.504

## Data Availability

Not applicable.
